# Scalar anomaly cancellation reveals the hidden superalgebraic structure of the quantum chiral SU(2/1) model of leptons and quarks

**DOI:** 10.1007/jhep10(2020)167

**Published:** 2020-10-26

**Authors:** Jean Thierry-Mieg

**Affiliations:** NCBI, National Library of Medicine, National Institute of Health, 8600 Rockville Pike, Bethesda MD20894, U.S.A.

**Keywords:** Anomalies in Field and String Theories, Gauge Symmetry, Beyond Standard Model

## Abstract

At the classical level, the SU(2/1) superalgebra offers a natural description of the elementary particles: leptons and quarks massless states, graded by their chirality, fit the smallest irreducible representations of SU(2/1). Our new proposition is to pair the left/right space-time chirality with the superalgebra chirality and to study the model at the one-loop quantum level. If, despite the fact that they are non-Hermitian, we use the odd matrices of SU(2/1) to minimally couple an oriented complex Higgs scalar field to the chiral Fermions, novel anomalies occur. They affect the scalar propagators and vertices. However, these undesired new terms cancel out, together with the Adler-Bell-Jackiw vector anomalies, because the quarks compensate the leptons. The unexpected and striking consequence is that the scalar propagator must be normalized using the antisymmetric super-Killing metric and the scalar-vector vertex must use the symmetric *d_aij* structure constants of the superalgebra. Despite this extraordinary structure, the resulting Lagrangian is actually Hermitian.

## 1 Background

The weak interactions are chiral. All the left helicity states of the leptons and the quarks are weak SU(2) doublets, whereas all their right helicity states are SU(2) singlets.

This fundamental asymmetry, first recognized by Lee and Yang in 1957, remains a challenge to the algebraic classification of the elementary particles because the Lie algebra Yang-Mills multiplets can only describe massless Fermions of a given chirality, hence cannot unify the different helicity states of the particles. Two avenues have been explored. On the one hand, in the grand unified theories, the anti right-singlets, which are left anti-singlets, are combined with the left doublets. As particles are coupled to antiparticles, the baryon number is not conserved and an observable slow decay rate of the proton is predicted. But this proton decay was not observed in dedicated experiments. On the other hand, in supersymmetric models, each known particle must be associated to a new particle: the s-electron, s-quark, gluino and so on. But the CERN hadron collider has revealed no new physics below 1Tev. Both approaches thus seem incompatible with experiments. With hindsight, these models did not mark ‘the end of physics’, and the door remains open to alternative ideas.

In 1979 Ne’eman [1] and Fairlie [2] proposed to embed SU(2)U(1) in the Lie-Kac superalgebra SU(2*/*1). Their paradigm is to use the chirality *χ* as the fundamental *Z*(2) grading of the superalgebra [3], allowing the unification of left and right Fermion states in graded multiplets. The germ of this idea can be traced back to the original presentation of the electroweak unification by Weinberg in 1967 [4] where he noticed that since there is no massless particle coupled to the electron number, the U(1) gauge field must be proportional to the electronic hypercharge $Y=N_{R}+N_{L}/2$ which has the same trace over the left and the right leptons

(1.1) $$Tr_{L}(Y)-Tr_{R}(Y)=Tr(\chi Y)=STr(Y)=0,$$

precisely the condition allowing to embed SU(2)U(1) inside SU(2*/*1).

The cancellation of the Adler-Bell-Jackiw anomaly [5, 6],

(1.2) $$C_{abc}=STr\left(\lambda_{a},{\left\{\lambda_{b},\lambda_{c}\right\}}_{+}\right)=0$$

gives another indication as it involves a supertrace and an anticommutator and corresponds to the even part of the cubic super-Casimir tensor of SU(2*/*1) (appendix A equation (A.7) and appendix I equation (I.5)).

At the static classification level, the SU(2*/*1) model is successful. The leptons [1, 2], the quarks [7, 8], and their antiparticles are naturally described (appendix B, C and D) by the lowest dimensional SU(2*/*1) irreducible representations [3, 9], unifying in the same chiral multiplets the left and the right massless states. In addition, contrary to Lie algebras, superalgebras admit finite dimensional indecomposable representations [10–12], which in the case of SU(2*/*1) can regroup at most three generations of quarks (appendix H and [13–16]).

In other words, SU(2*/*1) offers an ideal algebraic classification of all the existing fundamental Fermions: unlike GUTs SU(2*/*1) does not predict proton decay, unlike SUSY SU(2*/*1) does not require the existence of new Fermions, yet SU(2*/*1) is the only algebraic model which naturally predicts the number of generations of leptons and quarks.

The symmetry breaking pattern of the adjoint representation is also satisfactory. Following Yang and Mills, the SU(2)U(1) even generators are gauged by the *W*^±^, the *Z*^0^ and the photon. We then postulate that scalar fields correspond to the odd generators. So if they acquire a non-zero vacuum expectation value *v*, then *v* selects one of the odd directions. But since in a superalgebra the odd generators close by anticommutation on the even generators, the square *γ* = {*v,v*}_+_ of the vacuum automatically corresponds to an even generator that we can identify as the photon. The super-Jacobi identity (appendix A, equation (A.4)) then implies that the photon commutes with *v*:

(1.3) $$[\gamma ,v]={\left[{\left\{v,v\right\}}_{+},v\right]}_{-}=0$$

and remains massless [8, 17, 18]. In 1995, using SU(2*/*1) in a qualitative way, Hwang, Lee and Ne’eman [19] correctly predicted the mass of the Higgs to be 130 ± 6Gev, seventeen years before the experimental observation at 125Gev.

The difficulty in the SU(2*/*1) model is to extend the Lie algebra Yang-Mills formalism to the more complex case of a superalgebra. Our proposition is to bypass the construction of the classical theory and directly study the Fermions quantum one-loop counterterms which can be computed just from the assumption that the Bosons are coupled to the Fermions according to the matrices *λ* of the relevant linear representation of the SU(2*/*1) superalgebra. This has never been attempted, probably because the quarks odd matrices, listed in appendix F, are not Hermitian. Analyzing the scalar propagator and vertices counter-terms, we show below that because our new scalar-Fermions couplings are non Hermitian and chiral, the counterterms contain a regular part and an anomaly. Our surprising discovery is that, exactly like in the Adler-Bell-Jackiw triangle diagrams, the sum of the lepton and quark contributions [20] cancels out these new scalar anomalies, whereas, as shown in equation (2.9), the regular counterterms induce a scalar Lagrangian

(1.4) $$\begin{array}{c} L_{\text{Φ}}=-g_{ij}D_{\mu }{\bar{\text{Φ}}}^{i}D^{\mu }{\text{Φ}}^{j}.\\ g_{ij}=\frac{1}{2}STr\left(\lambda_{i}\lambda_{j}\right),\;D_{\mu }{\text{Φ}}_{i}=\partial_{\mu }{\text{Φ}}_{i}+d_{aij}A_{\mu }^{a}{\text{Φ}}^{j}. \end{array}$$

exactly as expected of a minimally coupled superalgebra, where the normalization of the scalar propagator is proportional to the *g*_*ij*_ super-Killing metric and the regular vector-scalar counterterm is proportional to the *d*_*aij*_ symmetric structure constants of SU(2*/*1). Despite this unusual structure, the theory is unitary because a linear change of variables given in equation (3.1) leads back to a classic Lie algebra Hermitian Lagrangian.

In other words, we are not constructing a locally supersymmetric version of the standard model, but we reveal, at the quantum dynamical level, the existence inside the model of several new hidden layers of SU(2*/*1) superalgebraic structures.

In the following sections, we present our new results. But since we realize that the SU(2*/*1) model is not well known, we recall in the appendices the definition of a chiral superalgebra, the construction of the leptons and quarks SU(2*/*1) irreducible or indecomposable representations, and the principal steps in the calculation of the Adler-Bell-Jackiw vector anomaly.

## 2 The chiral scalar-Fermion minimal coupling

Let us assume the existence of an oriented complex scalar field $\bar{\text{Φ}}\text{Φ}$ coupled to the chiral Fermions $\bar{\psi }\psi$ via the odd generators *λ*_*i*_ of the superalgebra The scalars are oriented: they transport left spin states, they are emitted by left *ψ*_*L*_ Fermions (which then become right) and absorbed by right *ψ*_*R*_ Fermions (which then become left) according to the Feynman diagrams:

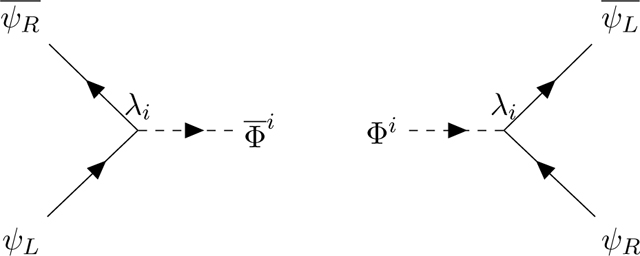


To preserve CP invariance, we need to multiply the odd matrices *λ*_*i*_ by a chiral projector

(2.1) $$\epsilon_{L}=\frac{1}{2}(1+\chi ),\;\epsilon_{R}=\frac{1}{2}(1-\chi ).$$

The chirality operator *χ*, which acts on the algebra charges and defines the supertrace (appendix A, equation (A.1)), is correlated with the Lorentz chirality operator *γ*_5_, which acts on the spin indices. Φ is absorbed by an SU(2) singlet right-spinor $\psi_{R}=\frac{1}{4}(1-\chi )\left(1+\gamma_{5}\right)$, emitting an SU(2) doublet left-spinor $\psi_{L}=\frac{1}{4}(1+\chi )\left(1-\gamma_{5}\right)$. This correlation explains why the weak interactions break *C* and *P* but conserve *CP*. There is no equivalent relation in the Yang-Mills-Lie algebra framework because the charge chirality *χ* is specific of superlagebras. The Fermion-scalar interaction terms of the Lagrangian read:

(2.2) $$L_{\psi \text{Φ}}={\bar{\left(\psi_{L}\right)}}_{R}{\text{Φ}}^{i}\epsilon_{L}\lambda_{i}\psi_{R}+{\bar{\left(\psi_{R}\right)}}_{L}{\bar{\text{Φ}}}^{i}\epsilon_{R}\lambda_{i}\psi_{L}.$$

For the moment, we do not specify the Lagrangian of the Φ scalars. The idea is to deduce the nature of the propagator of the scalars and their interactions with the vector fields from the calculation of the Fermion loops. Consider first the propagator:

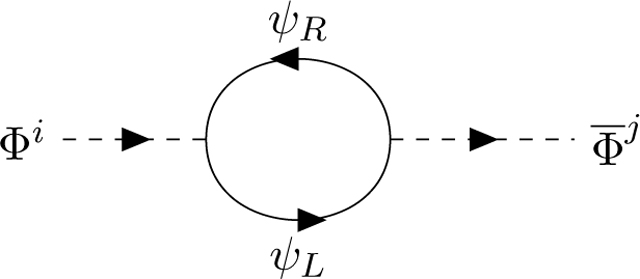


This counterterm is, as it should, proportional to the inverse square of the momentum *p* of the propagating scalar (1*/p*^2^), but the trace over the odd matrices is chiral. We get

(2.3) $$Tr\left(\epsilon_{L}\lambda_{i}\lambda_{j}\right)=\frac{1}{2}STr\left(\lambda_{i}\lambda_{j}\right)+\frac{1}{2}Tr\left(\lambda_{i}\lambda_{j}\right)$$

We like the first term which gives the odd part of the super-Killing metric of the superalgebra. The second term gives the ‘would be’ symmetric metric of a Lie algebra, but is not an invariant of a superalgebra. Generalizing the Adler-Bell-Jackiw condition (1.2), we call it anomalous and request that the combined contribution of all chiral Fermions vanishes:

(2.4) $$Tr\left(\lambda_{i}\lambda_{j}\right)=0.$$

We now consider the scalar-scalar-vector triangle diagram. There are only two diagrams corresponding to the two possible orientations of the Fermion loop, versus the four diagrams shown in appendix I in the case of the vector anomaly. Since the Fermion loop absorbs ${\text{Φ}}^{i}$ and emits ${\bar{\text{Φ}}}^{j}$ the orientation of the loop imposes the chirality.


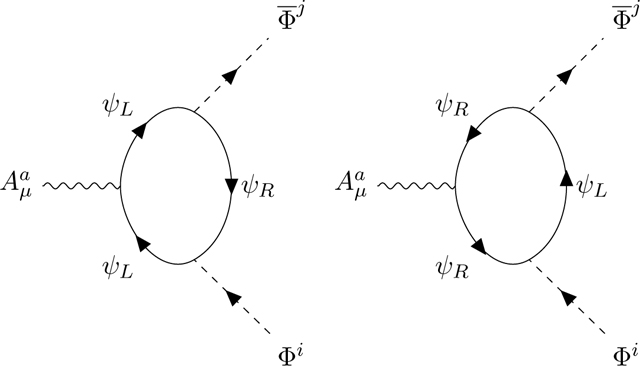


In one orientation, the vector $A_{\mu }^{a}$ touches a left Fermion, in the opposite orientation it touches a right Fermion, and as recalled in appendix I for the Adler-Bell-Jackiw triangle diagram, the orientation governs the overall sign of the diagram. Hence we obtain the unusual term

(2.5) $$Tr\left(\epsilon_{L}\lambda_{a}\lambda_{i}\lambda_{j}-\epsilon_{R}\lambda_{a}\lambda_{j}\lambda_{i}\right)=\frac{1}{2}STr\left(\lambda_{a}{\left\{\lambda_{i},\lambda_{j}\right\}}_{+}\right)+\frac{1}{2}Tr\left(\lambda_{a}{\left[\lambda_{i},\lambda_{j}\right]}_{-}\right).$$

The first term of (2.5) gives, for any representation of the superalgebra, the symmetric structure constants of the superalgebra (appendix A, equations (A.3) and (A.6)):

(2.6) $$d_{aij}=\frac{1}{2}STr\left(\lambda_{a}{\left\{\lambda_{i},\lambda_{j}\right\}}_{+}\right)$$

The second term of (2.5) gives the ‘would be’ antisymmetric constants

(2.7) $$f_{aij}=\frac{1}{2}Tr\left(\lambda_{a}{\left[\lambda_{i},\lambda_{j}\right]}_{-}\right)$$

which are not well defined, because the commutators of the odd matrices do not close on the even matrices. We call this second term anomalous, and generalizing the Adler-Bell-Jackiw condition (1.2) we request that:

(2.8) $$Tr\left(\lambda_{a}{\left[\lambda_{i},\lambda_{j}\right]}_{-}\right)=0.$$

Our surprising result is that the three conditions (1.2), (2.4) and (2.8) are met simultaneously when we apply the experimentally validated Bouchiat-Iliopoulos-Meyer prescription: 3 quarks for every lepton [20]. In other words, the propagator (2.4) and vertex (2.8) scalar anomalies vanish, provided the Adler-Bell-Jackiw anomaly (1.2) vanishes. The 3 conditions are verified by direct examination of the quark and lepton matrices listed in appendix B and D. The three anomalies also vanish if we consider the antileptons and antiquarks matrices listed in appendix C and E.

Therefore, the renormalization rules (2.3), (2.5) imply that the Lagrangian of the scalar field is explicitly supercovariant:

(2.9) $$L_{\text{Φ}}=-g_{ij}D_{\mu }{\bar{\text{Φ}}}^{i}D_{\mu }{\text{Φ}}^{j},\;D_{\mu }{\text{Φ}}_{i}=\partial_{\mu }{\text{Φ}}_{i}+d_{aij}A_{\mu }^{a}{\text{Φ}}^{j},$$

where *g*_*ij*_ is the antisymmetric super-Killing metric (appendix A, equation (A.5)) and the supercovariant derivative *D*_*μ*_ produces the (*ij*) vertex *d*_*aij*_ (*p* + *q*)_*μ*_ where the *d*_*aij*_ are the symmetric structure constants of the superalgebra (appendix A, equation (A.3)), and p and q are the momenta of the incoming and outgoing Φ fields in the orientation of the Φ lines.

Finally, we consider the $AA\bar{\text{Φ}}\text{Φ}$ two-vectors-two-scalars vertex which gives an additional constraint.


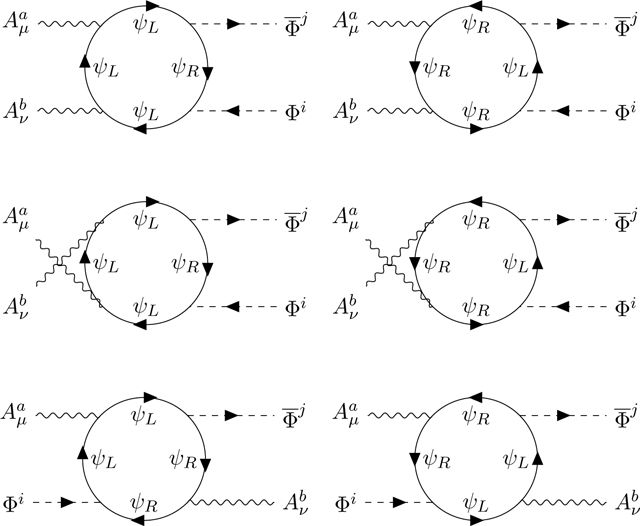


The diagrams are symmetrized in (*aμ,bν*) but not in (*ij*) since Φ and $\bar{\text{Φ}}$ are distinct. Carefully computing the trace of six *σ* matrices (appendix I, equations (I.3)–(I.4)), we find that the counterterm is proportional to

(2.10) $$Tr\left(\left(\lambda_{a}\lambda_{b}+\lambda_{b}\lambda_{a}\right)\left(\epsilon_{L}\lambda_{i}\lambda_{j}+\epsilon_{R}\lambda_{j}\lambda_{i}\right)-2\left(\epsilon_{L}\lambda_{a}\lambda_{i}\lambda_{b}\lambda_{j}+\epsilon_{R}\lambda_{a}\lambda_{j}\lambda_{b}\lambda_{i}\right)\right).$$

This trace can be decomposed into the sum of two terms

(2.11) $$g^{ij}\left(d_{aik}d_{bjl}+d_{bik}d_{ajl}\right)+\text{Δ}(\rho )\delta_{ij}\left(f_{ak}^{i}f_{bl}^{j}+f_{bk}^{i}f_{al}^{j}\right).$$

We like the first term of this equation. It is proportional to (*d*_*a..*_)^2^ which is characteristic of a superalgebra. It is representation independent. It matches the term $g^{\mu \nu }A_{u}^{a}A_{\nu }^{b}{\bar{\text{Φ}}}^{k}{\text{Φ}}^{l}g^{ij}\left(d_{aik}d_{bjl}+d_{bik}d_{ajl}\right)$ present in the classical Lagrangian (2.9). Therefore, it can be absorbed by a renormalization of the coupling constant g^2^. The relative renormalization of g in the $\bar{\text{Φ}}\text{Φ}$, $\text{g}A\bar{\text{Φ}}\text{Φ}$ and ${\text{g}}^{2}AA\bar{\text{Φ}}\text{Φ}$ diagrams is correct because the integrals over the loop-momenta are the same as in the standard Yang-Mill-scalar theory, only the group traces are new. The second term of (2.11) is proportional to (*f*_*a..*_)^2^ which is characteristic of a ‘would be’ Lie algebra. Its normalization Δ(*ρ*) depends on the representation. We call this term anomalous and verified numerically, with a simple C-program, that the combined quark and lepton contributions again cancel out thanks to the BIM mechanism [20]

(2.12) Δ( leptons )≠0,Δ( leptons )+3Δ( quarks )=0.

In conclusion, the $AA\bar{\text{Φ}}\text{Φ}$ term is renormalizable, establishing a new scalar generalization of the Ward, Takahashi, Slavnov, Taylor identity to the case of the SU(2*/*1) superalgebra.

As shown at the end of appendix E, any combination of leptons and quark-like representations such that the total sum of the hypercharges of the left doublets vanishes is anomaly free. We already discussed the standard model assignment, one electron of hypercharge −1 and 3 colors of quarks of hypercharge 1*/*3, but we could also consider the OSp(2*/*1) neutral representation of Minahan, Ramon and Warner (appendix E and [21]), or one quark doublet of hypercharge 2*/*3 and two of hypercharge −1*/*3, and so on. We leave as an open problem the general classification of all the chiral representations of the simple superalgebras satisfying the four equations (1.2), (2.4), (2.8), (2.12) and conjecture that these anomalies play a role in the exponentiation of the superalgebra into a supergroup.

These results are unexpected and were not anticipated in the SU(2*/*1) literature. It was known since the early eighties that the quantum numbers of quarks and leptons corresponded to the SU(2*/*1) irreducible representations, but there was no sign that the superalgebra metric and the *d*_*aij*_ superstructure constants could play a role in the dynamics of the theory.

A vertex proportional to the *d*_*aij*_ symmetric structure constant is actually a necessity in a superalgebraic theory. Consider the renormalization of the vector-Fermion vertex where the vector $A_{\mu }^{a}$ emits a pair ${\bar{\text{Φ}}}^{i}{\text{Φ}}^{j}$ via a vertex *h*_*aij*_ with unknown (*ij*) symmetry. The 2 scalars then hit the Fermion generating a matrix product *h*_*aji*_ *λ*_*i*_*λ*_*j*_:

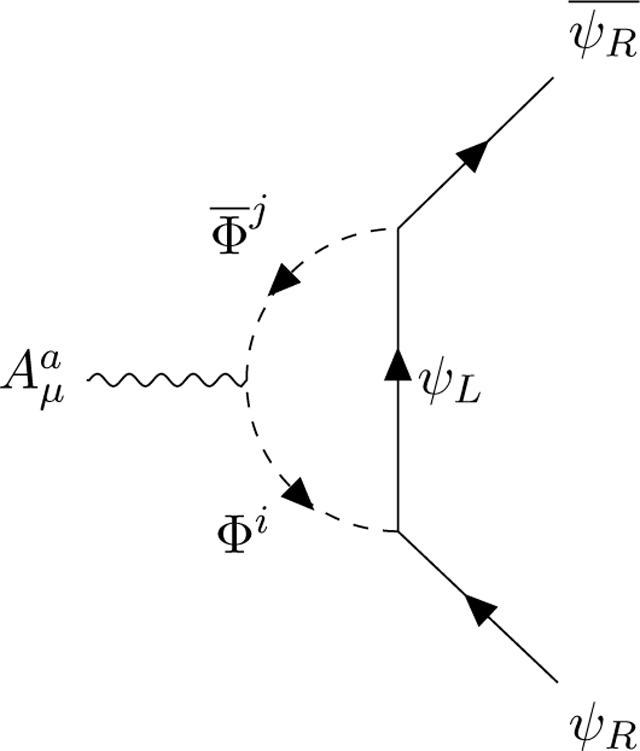


In the classic Yang-Mills case, the vector scalar vertex *f*_*aij*_ is antisymmetric in (*ij*) generating the commutator *f*_*aij*_ [*λ*_*i*_*λ*_*j*_] which closes on *λ*_*a*_. But in a superalgebra, we need an anticommutator, so *h*_*aij*_ has to be symmetric in (*ij*) and coincides with *d*_*aij*_. With Yuval Ne’eman, we were already hoping to solve this difficulty in 1982 by representing the odd generators using higher forms [22, 23], but that method did not produce the desired effect. This is why, after all these years, I am so pleased and so surprised by the new concept of the scalar anomaly cancellation presented here. The solution lies beyond the analysis of the abstract superalgebra structure and even beyond the analysis of its irreducible representation. It comes from the conspiracy of quarks and leptons. Separately, they each generate an anomaly, yet together they produce the desired symmetric vertex.

## 3 Rediagonalization to an explicitly Hermitian Lagrangian

From the analysis of the scalar anomalies, we found a very unusual structure for the covariant propagator of the Φ scalars (2.9). It involves the antisymmetric super-Killing metric *g*_*ij*_ and a *d*_*aij*_ symmetric structure constant in the definition of the covariant derivative. In addition, the scalar-Fermion vertex involves the non Hermitian odd matrices *λ*_*i*_.

To show that this theory is nevertheless consistent, we define new scalar fields *H* and *K* by the linear equations

(3.1) $$\begin{array}{l} {\text{Φ}}^{4}=\frac{1}{2}\left(H^{4}-iH^{5}+K^{5}-iK^{4}\right),\\ \begin{array}{c} {\text{Φ}}^{5}=\frac{1}{2}\left(H^{5}+iH^{4}-K^{4}-iK^{5}\right),\\ {\bar{\text{Φ}}}^{4}=\frac{1}{2}\left(H^{4}+iH^{5}-K^{5}-iK^{4}\right), \end{array}\\ \begin{array}{c} {\bar{\text{Φ}}}^{5}=\frac{1}{2}\left(-iH^{4}+H^{5}+K^{4}-iK^{5}\right),\\ {\text{Φ}}^{6}=\frac{1}{2}\left(H^{6}-iH^{7}+K^{7}-iK^{6}\right), \end{array}\\ \begin{array}{c} {\text{Φ}}^{7}=\frac{1}{2}\left(H^{7}+iH^{6}-K^{6}-iK^{7}\right),\\ {\bar{\text{Φ}}}^{6}=\frac{1}{2}\left(H^{6}+iH^{7}-K^{7}-iK^{6}\right),\\ {\bar{\text{Φ}}}^{7}=\frac{1}{2}\left(-iH^{6}+H^{7}+K^{6}-iK^{7}\right), \end{array} \end{array}$$

By substitution, we find that the super-Killing antisymmetric propagator (2.9) of the oriented Φ fields can be rewritten as a standard positive-defined diagonal propagator for the *H* and *K* scalar fields

(3.2) $$g_{ij}\partial^{\mu }{\bar{\text{Φ}}}^{i}\partial_{\mu }{\text{Φ}}^{j}=-\frac{1}{2}\delta_{ij}\left(\partial^{\mu }H^{i}\partial_{\mu }H^{j}+\partial^{\mu }K^{i}\partial_{\mu }K^{j}\right).$$

The couplings of the scalars to the Yang-Mills vectors also become standard. By substitution, we find that the unusual superalgebraic *d*_*aij*_ symmetric vertex (2.9) reverts to SU(2)U(1) minimal couplings

(3.3) $$d_{aij}A_{\mu }^{a}\left(\partial^{\mu }{\bar{\text{Φ}}}^{i}{\text{Φ}}^{j}+{\text{Φ}}^{i}\partial^{\mu }{\bar{\text{Φ}}}^{j}\right)=f_{aij}A_{\mu }^{a}\left(H^{i}{\overset{↔}{\partial }}^{\mu }H^{j}+K^{i}{\overset{↔}{\partial }}^{\mu }K^{j}\right)$$

Even more surprising, the non Hermitian couplings of Φ scalars to the chiral Fermions (1.4), (2.2) mutate into Hermitian couplings of the *H* and *K* scalars

(3.4) $$\epsilon_{R}{\bar{\text{Φ}}}^{i}\lambda_{i}+\epsilon_{L}{\text{Φ}}^{i}\lambda_{i}=H^{i}\mu_{i}^{-}+K^{i}\mu_{i}^{+}$$

where the $\mu_{i}^{-}$ matrices correspond to the Hermitian part of the *λ*_*i*_ matrices, and therefore interact only with the doublets and the negatively charged right Fermions singlets, and the $\mu_{i}^{+}$ matrices correspond to the anti-Hermitian part of the *λ*_*i*_ matrices, and therefore interact only with the doublets and the positively charged right Fermions singlets. In the lepton representation (appendix B)

(3.5) $$\mu_{i}^{-}=\lambda_{i},\;\mu_{i}^{+}=0,\;i=4,5,6,7$$

In the quark representation (appendix D)

(3.6) $$\mu_{6}^{-}=\frac{1}{\sqrt{3}}\left(\begin{array}{cccc} 0 & 0 & 0 & 0\\ 0 & 0 & 0 & 0\\ 0 & 0 & 0 & 1\\ 0 & 0 & 1 & 0 \end{array}\right),\;\mu_{6}^{+}=\frac{1}{\sqrt{3}}\left(\begin{array}{cccc} 0 & -i\sqrt{2} & 0 & 0\\ i\sqrt{2} & 0 & 0 & 0\\ 0 & 0 & 0 & 0\\ 0 & 0 & 0 & 0 \end{array}\right).$$

The other odd matrices *μ*_4_,*μ*_5_,*μ*_7_ follow the same pattern and are given in appendix F.

The *H* and *K* fields have been defined previously by Haussling and Scheck in [15, 16], but without proper justification. Noticing that the odd quark matrices *λ*_*i*_ are non Hermitian, they added to the natural scalar-Fermion Lagrangian (2.2) its Hermitian conjugate $L^{†}$, in a way double-counting the particles and the antiparticles. This induced the same Hermitian scalar-Fermion coupling $H^{i}\mu_{i}^{-}+K^{i}\mu_{i}^{+}$ (3.4)–(3.6), but they could not relate *H* and *K* to $\bar{\text{Φ}}\text{Φ}$ because they implicitly assumed that the $\bar{\text{Φ}}\text{Φ}$ Lagrangian has the usual structure $L=\delta_{ij}D^{\mu }{\bar{\text{Φ}}}^{i}D_{\mu }{\text{Φ}}^{j}$ with $D_{\mu }{\text{Φ}}^{j}=\partial_{\mu }{\text{Φ}}^{j}+f_{ak}^{j}{\text{Φ}}^{k}$.

In conclusion, using an axiomatic top-down approach, we have discovered that the ‘Standard Model’ equipped with a conventional complex Higgs scalar SU(2) doublet *H*+*iK* hides an explicit superalgebraic structure, which is revealed by rewriting the *H* and *K* fields in terms of the superalgebraic $\bar{\text{Φ}}$ and Φ fields using the linear equation (3.1). Furthermore, if we start from the antileptons and antiquarks representations, we find exactly the same *H K* Lagrangian. These transformations only make sense in the quantum world and are implied by the analysis of the anomalies of the one-loop counterterms.

## 4 Generation mixing

The *H* and *K* fields bring us back to the study by Haussling and Scheck of the indecomposable representations of SU(2*/*1) [13, 15, 16]. Since these representations can be written as block triangular matrices (appendix G and H), the mixing terms do not contribute to the calculation of matrix traces, so they do not modify our calculation of the anomalies (1.2), (2.4), (2.8), (2.12). Therefore, the indecomposable representations of SU(2/1) are admissible and lead to the same definition (3.1) of the *H* and *K* fields.

These representations provide, inside the SU(2/1) framework, a natural uinderstanding of neutrino oscillations ([16] and appendix G), and of the existence of at most three generations of quarks and leptons with their mixing angles ([15] and appendix H), a schema that no other algebraic model explains. But since the mixing angles do not play a role in the calculations, the anomaly conditions do not link the leptons mixing angles to the quarks mixing angles. Note the direct contradiction with [18] which predicts 2^*p*^ generations.

This property of superalgebras overcomes an early counter argument of Feynman (private communication, 1979) who noticed that if the mass of the quarks could be explained by an irreducible symmetry, then the *up*, *charm* and *top* quarks would have the same mass. The solution of this paradox is that the SU(2*/*1) superalgebra admits a single indecomposable representation which describes at once the three generations explaining why the quarks have unequal masses and how heavier quarks decay into lighter quarks.

In contrast to the presentation of the Marseille-Mainz group [13–17], we believe that all these extraordinary results are direct consequences of the algebraic properties of the SU(2*/*1) superalgebra, and are not related in an obvious way to the non-commutative geometry of Alain Connes [24–26].

## 5 Limitations of the model

There remains an important problem in the construction of a fully consistent SU(2*/*1) quantum field theory. Contrary to the vector-Fermion vertex, the scalar-Fermion vertex is not protected by the Ward identities. Therefore the strong interactions contribute to the renormalization of the scalar-quark vertex although they do not affect the scalar-lepton vertex. As a result, the balance between the leptons and the quarks necessary to cancel the scalar anomalies does not seem to be preserved at the 2-loop level. An open problem is to see if this is a genuine obstruction, and if so, can the symmetry be restored, for instance by incorporating aspects of the non commutative differential geometry of Connes [25], or the self-dual scalars of Avdeev-Chizhov [27, 28], or OSp(4/2) Fermion ghosts [29], or any new idea.

## 6 Discussion

The weak interactions are chiral. Before symmetry breaking, the leptons and quarks are massless, their left and right helicity states are distinct, and only the left states couple to the weak SU(2) interactions. As understood by Weinberg [4] in 1967, there are no charged massless Fermions, so the total hypercharge *Y* of the left and right states must be equal: $Tr_{L}(Y)-Tr_{R}(Y)=Tr(\chi Y)=STr(Y)=0$, allowing to identify the electroweak SU(2)U(1) Lie algebra with the even part of the Kac superalgebra SU(2*/*1), graded by chirality [1, 2].

The same conclusion can be derived from the study of Adler-Bell-Jackiw anomaly [5, 6]. Applied to the U(1)SU(3)SU(3) quark loop, we learn that *STr*(*Y* ) = 0 for the quarks. Applied to the U(1)SU(2)SU(2) Fermion loop, we learn, as discovered by Bouchiat Iliopoulos and Meyer [20], that the lepton and the quark diagrams are both anomalous, but the lepton loop is compensated by three quark loops (BIM mechanism). Furthermore, the Adler Bell-Jackiw anomaly (1.2) is proportional to the even part of the cubic super-Casimir tensor of SU(2*/*1) (appendix A, equation (A.7)).

The purpose of our study is to cast the three families of leptons and quarks into representations of the SU(2*/*1) superalgebra and to associate the Higgs field to the odd generators. This idea was first proposed independently in 1979 by Ne’eman [1] and Fairlie [2] who observed, as shown in appendix B, that the (2/1) fundamental representation of SU(2*/*1) fits the leptons (*ν*_*l*_,*e*_*L*_*/e*_*R*_) graded by their chirality. The model was rapidly extended to the quarks (*u*_*R*_*/u*_*L*_,*d*_*L*_*/d*_*R*_) by Dondi, Jarvis, Ne’eman and Thierry-Mieg [7, 8] which, as shown in appendix D, fit the smallest typical representation of SU(2*/*1) [9]. On the lepton side, as shown in appendix E, SU(2*/*1) specifies that if the charge of the *e*^−^ electron is equal to the charge of the *W*^−^ vector Boson, then the U(1) charge of the right neutrino vanishes [8]. Hence the right neutrino should be weakly neutral, an experimentally validated prediction. It was then discovered in the nineties [13–17] that the indecomposable representations of SU(2*/*1) fit the existence and decays of the heavier families.

A main perceived problem of the SU(2*/*1) model is that the odd matrices are non Hermitian. For example, one can choose a base where the electron odd matrices are Hermitian (appendix B), but since the square of the matrix *λ*_6_ gives the electric charge, it follows that in the antielectron representation, (*λ*_6_)^2^ has the opposite sign, hence the odd antielectron matrices are anti-Hermitian (appendix C). Furthermore, the quark and antiquark odd matrices are neither Hermitian nor anti-Hermitian (appendix D). This complexity seemed to prevent any form of minimal coupling.

But here, we report a discovery. If we strictly apply the SU(2*/*1) representation theory and associate the odd generators of SU(2*/*1) to an oriented complex doublet of scalar (Higgs) fields coupling the left and right Fermions, the non Hermitian character of the odd matrices generates a new set of anomalies. The one-loop leptons or quarks contributions to the self diffusion of the vector Bosons (1.2), to the propagator of the scalars (2.4), and to the diffusion of the scalars by the vector Bosons (2.8) are all anomalous. However, the contributions of the leptons are exactly compensated by those of the three quarks [20], canceling at the same time the Adler-Bell-Jackiw vector anomalies [5, 6] and the new scalar anomalies discovered here. It follows that the propagator of the complex scalars is given by the odd part of the super-Killing metric of SU(2*/*1) and that the $A_{\mu }^{a}{\text{Φ}}^{i}{\text{Φ}}^{j}$ coupling is given by the *d*_*aij*_ symmetric structure constant characteristic of a superalgebra (2.9). We also establish a superalgebraic scalar Ward identity (2.12) linking the renormalization of the $\bar{\text{Φ}}\text{Φ}$ propagator, $A\bar{\text{Φ}}\text{Φ}$ triangle diagram and $AA\bar{\text{Φ}}\text{Φ}$ square diagram, as another new consequence of the BIM mechanism. All calculations were done manually and verified numerically using a simple C-language program. A linear change of variables (3.1) then transforms back this unusual Lagrangian to a classic model with a pair of scalars *H* and *K* respectively coupled to the up and down right quark states, *u*_*R*_ and *d*_*R*_, via Hermitian matrices (3.5)–(3.6), without breaking the algebraic structure by artificially adding the Hermitian conjugated Lagrangian as was necessary in [13–17].

Although SU(2*/*1) is a superalgebra, the present construction respects the statistics of the particles: the Yang-Mills vectors and the scalars are commuting Bosons, the leptons and quarks are spin-half anticommuting Fermions, and all interactions are Lorentz covariant. Rather than changing Bosons into Fermions like the Wess-Zumino space-time supersymmetry, the SU(2*/*1) internal supersymmetry exchanges the chirality of the Fermions without changing their statistics. Furthermore, the pairing (2.1) of the left/right space-time chirality *γ*_5_, with the charge chirality *χ* which defines the supertrace of the superalgebra, provides an algebraic explanation of the *CP* structure of the weak interaction which is lacking in the classic Yang-Mills Lie algebra formalism.

In conclusion, we recall that the SU(2)U(1) standard model of the electroweak interactions contains a hidden chiral SU(2*/*1) superalgebraic structure [1, 2] which explains the quantum numbers of the quarks [7, 8] using non-Hermitian matrices. The necessary cancellation of the resulting scalar anomalies dictates the structure of the scalar Lagrangian, and we have for the first time established a new kind of minimal coupling of a chiral superalgebra where the Hermitian Lie subalgebra matrices define as usual the emission/absorption of the Yang-Mills vector Bosons by the Fermions, and where the non-Hermitian odd generators define the chirality flipping absorption/emission of an oriented scalar Higgs field by the chiral Fermions. In this framework, the super-Killing metric and the *d*_*aij*_ superstructure constants of SU(2*/*1) define the propagator and vector diffusions of the chirality aware Higgs scalars, which naturally complement the Yang-Mills field in the intrinsic-geometrical definition of the Lie superalgebra chiral connection [30].

## A Definition of a chiral superalgebra

Let us define, using the notations of [30], a chiral-superalgebra as a finite dimensional basic classical Lie-Kac superalgebra [3], graded by chirality. For example, we could take a super-algebra of type SU(*m/n*), or OSp(*m/*2*n*) or a product of Lie algebras and superalgebras like the SU(2*/*1) superalgebra of the standard model.

The superalgebra acts on a finite dimensional space of massless Fermion states graded by their helicity. The chirality matrix *χ* is diagonal, with eigenvalue 1 on the left Fermions and −1 on the right Fermions. It defines the supertrace

(A.1) STr(…)=Tr(χ…)

Each generator is represented by a finite dimensional matrix of complex numbers (we do not need anticommuting Grassman numbers). The even generators are denoted *λ*_*a*_ and the odd generators *λ*_*i*_. *χ* commutes with the *λ*_*a*_ and anticommutes with the *λ*_*i*_

(A.2) $${\left[\chi ,\lambda_{a}\right]}_{-}={\left\{\chi ,\lambda_{i}\right\}}_{+}=0$$

The *λ* matrices close under (anti)-commutation

(A.3) $${\left[\lambda_{a},\lambda_{b}\right]}_{-}=f_{ab}^{c}\lambda_{c},\;{\left[\lambda_{a},\lambda_{i}\right]}_{-}=f_{ai}^{j}\lambda_{j},\;{\left\{\lambda_{i},\lambda_{i}\right\}}_{+}=d_{ij}^{a}\lambda_{a},$$

and satisfy the super-Jacobi relation with 3 cyclic permuted terms:

(A.4) $${\left(-1\right)}^{AC}\text{\{}\lambda_{A},\text{\{}\lambda_{B},\lambda_{C}\text{]]}+{\left(-1\right)}^{BA}\text{\{}\lambda_{B},\text{\{}\lambda_{C},\lambda_{A}\text{]]}+{\left(-1\right)}^{CB}\text{\{}\lambda_{C},\text{\{}\lambda_{A},\lambda_{B}\text{]]}=0.$$

The quadratic Casimir tensor (*g*_*ab*_,*g*_*ij*_), also called the super-Killing metric, is defined as

(A.5) $$\begin{array}{c} g_{ab}=\frac{1}{2}STr\left(\lambda_{a}\lambda_{b}\right),\\ g_{ij}=\frac{1}{2}STr\left(\lambda_{i}\lambda_{j}\right). \end{array}$$

The even part *g*_*ab*_ of the metric is as usual symmetric, but because the odd generators anticommute (A.2) with the chirality hidden in the supertrace (A.1), its odd part *g*_*ij*_ is antisymmetric. The structure constants can be recovered from the supertrace of a product of 3 matrices

(A.6) $$\begin{array}{c} f_{abc}=g_{ae}f_{bc}^{e}=\frac{1}{2}STr\left(\lambda_{a}{\left[\lambda_{b},\lambda_{c}\right]}_{-}\right),\\ d_{aij}=g_{ae}d_{ij}^{e}=\frac{1}{2}STr\left(\lambda_{a}{\left\{\lambda_{i},\lambda_{j}\right\}}_{+}\right), \end{array}$$

The cubic Casimir tensor is defined as

(A.7) $$\begin{array}{c} C_{abc}=\frac{1}{2}STr\left(\lambda_{a}{\left\{\lambda_{b},\lambda_{c}\right\}}_{+}\right),\\ C_{aij}=\frac{1}{2}STr\left(\lambda_{a}{\left[\lambda_{i},\lambda_{j}\right]}_{-}\right). \end{array}$$

The Casimirs use the ‘wrong’ type of commutator, otherwise, using equation (A.3), they could be simplified. We have $g_{ai}=C_{abi}=C_{ijk}=0$ since the diagonal elements of the product of an odd number of odd matrices necessarily vanish. Using these tensors, we can construct the super-Casimir operators

(A.8) $$K_{2}=g^{AB}\lambda_{A}\lambda_{B},\;K_{3}=C^{ABC}\lambda_{A}\lambda_{B}\lambda_{C},$$

where the upper index metric *g*^*AB*^ is the inverse of the lower metric *g*_*AB*_, summation over the repeated indices is implied and ranges over even and odd values *A, B* = *a,b, . . . ,i,j . . .*, and the indices of *C*^*ABC*^ are raised using *g*^*AB*^. The Casimir operators *K*_2_ and *K*_3_ commute with all the generators of the superalgebra. In an irreducible representation, they are represented by a multiple of the identity matrix. In SU(2*/*1), which has rank 2, they form a basis of its enveloping superalgebra.

## B The SU(2/1) lepton representation

Consider the left neutrino and the left and right electron states collectively called the leptons (*ν*_*L*_,* e*_*L*_ */ e*_*R*_). Their experimentally observed chirality and weak hyper-charge are given by the diagonal matrices

(B.1) $$\chi =\left(\begin{array}{ccc} 1 & 0 & 0\\ 0 & 1 & 0\\ 0 & 0 & -1 \end{array}\right),\;\lambda_{0}=\left(\begin{array}{ccc} -1 & 0 & 0\\ 0 & -1 & 0\\ 0 & 0 & -2 \end{array}\right).$$

With respect to the chiral *Z*_2_ grading, the supertrace of *λ*_0_ vanishes:

(B.2) $$STr\left(\lambda_{0}\right)=Tr\left(\chi \lambda_{0}\right)=0.$$

This is the first indication that the electroweak interactions could be described by a super-algebra. In the same coordinates, the SU(2) weak charges are given by the matrices

(B.3) $$\lambda_{1}=\left(\begin{array}{ccc} 0 & 1 & 0\\ 1 & 0 & 0\\ 0 & 0 & 0 \end{array}\right),\;\lambda_{2}=\left(\begin{array}{ccc} 0 & -i & 0\\ i & 0 & 0\\ 0 & 0 & 0 \end{array}\right),\;\lambda_{3}=\left(\begin{array}{ccc} 1 & 0 & 0\\ 0 & -1 & 0\\ 0 & 0 & 0 \end{array}\right).$$

The four *λ*_*a*_ matrices (*a* = 0,1,2,3) represent the Lie algebra SU(2).U(1). They close under commutation

(B.4) $${\left[\lambda_{b},\lambda_{c}\right]}_{-}=f_{bc}^{a}\lambda_{a}\;a,b,c=0,1,2,3$$

and the only non zero structure constants are $f_{23}^{1}=f_{31}^{2}=f_{12}^{3}=2i$.

Let us now add in the picture the four Hermitian matrices

(B.5) $$\lambda_{4}=\left(\begin{array}{ccc} 0 & 0 & 1\\ 0 & 0 & 0\\ 1 & 0 & 0 \end{array}\right)\;\lambda_{5}=\left(\begin{array}{ccc} 0 & 0 & -i\\ 0 & 0 & 0\\ i & 0 & 0 \end{array}\right)\;\lambda_{6}=\left(\begin{array}{ccc} 0 & 0 & 0\\ 0 & 0 & 1\\ 0 & 1 & 0 \end{array}\right)\;\lambda_{7}=\left(\begin{array}{ccc} 0 & 0 & 0\\ 0 & 0 & -i\\ 0 & i & 0 \end{array}\right).$$

Under SU(2).U(1), these matrices have the same quantum numbers as the scalar Higgs doublet of the standard model, and they match the well known generators of SU(3). However, they do not close by commutations on the *λ*_*a*_, essentially because $Tr\left(\lambda_{0}\right)\neq 0$ (A.6). Rather (A.3), they close by anticommutation

(B.6) $${\left\{\lambda_{i},\lambda_{j}\right\}}_{+}=d_{ij}^{a}\lambda_{a}\;a=0,1,2,3\;i,j=4,5,6,7.$$

Observe also that they transform left leptons into right leptons and vice-versa, and therefore (A.2) anticommute with the chirality operator

(B.7) $${\left[\chi ,\lambda_{a}\right]}_{-}={\left\{\chi ,\lambda_{i}\right\}}_{+}=0\;a=0,1,2,3;\;i=4,5,6,7.$$

In this sense, the *λ*_*a*_ matrices are even, the *λ*_*i*_ are odd. Together they define the fundamental irreducible representation of the superalgebra SU(2*/*1), which appears first in Kac’s classification [3] under the name *A*(1*/*0).

With respect to the super-Killing metric (A.5) of SU(2*/*1)

(B.8) $$g_{AB}=\frac{1}{2}STr\left(\lambda_{A}\lambda_{B}\right)\;A,B=0,1\ldots 7$$

the even subspace has a Minkowski signature (−,+,+,+) and the electric charge operator

(B.9) $$\gamma =-\frac{1}{2}{\left\{\lambda_{6},\lambda_{6}\right\}}_{+}=\frac{1}{2}\left(\lambda_{0}+\lambda_{3}\right)=\left(\begin{array}{ccc} 0 & 0 & 0\\ 0 & -1 & 0\\ 0 & 0 & -1 \end{array}\right)$$

is on the light cone *STr*(*γ*^2^) = 0. In the terminology of Kac, the lepton representation is atypical and its two Casimir operators (A.8) vanish:

(B.10) $$K_{2}=K_{3}=0$$

This description of the SU(2*/*1) leptons was first proposed independently by Ne’eman and Fairlie in 1979 [1, 2, 18]. Its most remarkable feature is that it unifies in a single irreducible representation of the superalgebra the left and the right helicity state of the electron. This would be impossible in a Lie algebra where we would need to consider, as in the Georgi-Glashow grand-unified SU(5) model the left anti-(right electron) ${\bar{\left(e_{R}\right)}}_{L}$. A priori, the SU(2*/*1) symmetry could be exact at relatively low energy whereas the SU(5) grand-unification scale is necessarily extremely high to avoid a fast decay of particles into lighter antiparticles and avoid a contradiction with the observed stability of the proton.

## C The SU(2/1) antilepton representation

Except for *λ*_0_, the lepton matrices (B.1), (B.3), (B.5) look very familiar: they coincide with those of the fundamental 3 dimensional representation of SU(3). In particular, they are Hermitian. But this is a coincidence. If we turn to the antilepton representation, the electric charge of the positron is positive. As we must maintain the definition of the photon (B.9), the odd matrices must be anti-Hermitian. In the basis antielectron antineutrino: $\left({\left(\bar{e_{R}}\right)}_{L}/{\left(\bar{e_{L}}\right)}_{R},{\left(\bar{\nu_{L}}\right)}_{R}\right))$ the even matrices matrix read

(C.1) $$\lambda_{0}=\left(\begin{array}{ccc} 2 & 0 & 0\\ 0 & 1 & 0\\ 0 & 0 & 1 \end{array}\right),\;\lambda_{1}=\left(\begin{array}{ccc} 0 & 0 & 0\\ 0 & 0 & 1\\ 0 & 1 & 0 \end{array}\right),\;\lambda_{2}=\left(\begin{array}{ccc} 0 & 0 & 0\\ 0 & 0 & -i\\ 0 & i & 0 \end{array}\right),\;\lambda_{3}=\left(\begin{array}{ccc} 0 & 0 & 0\\ 0 & 1 & 0\\ 0 & 0 & -1 \end{array}\right),$$

and the 4 odd matrices read:

(C.2) $$\lambda_{4}=\left(\begin{array}{ccc} 0 & 0 & -1\\ 0 & 0 & 0\\ 1 & 0 & 0 \end{array}\right),\;\lambda_{5}=\left(\begin{array}{ccc} 0 & 0 & i\\ 0 & 0 & 0\\ i & 0 & 0 \end{array}\right),\;\lambda_{6}=\left(\begin{array}{ccc} 0 & 1 & 0\\ -1 & 0 & 0\\ 0 & 0 & 0 \end{array}\right),\;\lambda_{7}=\left(\begin{array}{ccc} 0 & -i & 0\\ -i & 0 & 0\\ 0 & 0 & 0 \end{array}\right).$$

The sign of Killing metric (A.5) flips because we must flip the chirality *χ*, but all the structure constants (A.3) are unchanged and the electric charge (B.9) of the positron reads

(C.3) $$\gamma =-\frac{1}{2}\left\{\lambda_{6},\lambda_{6}\right\}=-\frac{1}{2}\left\{\lambda_{7},\lambda_{7}\right\}=\frac{1}{2}\left(\lambda_{0}+\lambda_{3}\right)=\left(\begin{array}{ccc} 1 & 0 & 0\\ 0 & 1 & 0\\ 0 & 0 & 0 \end{array}\right)$$

The antilepton representation is also atypical, its two Casimir operators (A.8) again vanish:

(C.4) $$K_{2}=K_{3}=0.$$

Probably because they are anti-Hermitian, the odd matrices of the antilepton representation were never written explicitly in the SU(2*/*1) literature [18], although a complete theory of the anti-Fermions must be equivalent to a complete theory of the Fermions. Including both as in [13] would be over-counting. In reality, one cannot avoid facing these anti-Hermitian matrices since, as we shall see in the next section, the odd quark matrices are partly Hermitian and partly ant-Hermitian.

## D The SU(2/1) quark representation

Let us now consider the up and down quarks (*u*_*R*_ */* (*u*_*L*_,*d*_*L*_) */ d*_*R*_). They consist of an SU(2) left doublet and two right singlets with known weak hyper-charges 4*/*3,1*/*3*/*1*/*3,−2*/*3 and we can immediately write the even matrices.

(D.1) $$\chi =\left(\begin{array}{cccc} -1 & 0 & 0 & 0\\ 0 & 1 & 0 & 0\\ 0 & 0 & 1 & 0\\ 0 & 0 & 0 & -1 \end{array}\right),\;\lambda_{0}=\left(\begin{array}{cccc} 4/3 & 0 & 0 & 0\\ 0 & 1/3 & 0 & 0\\ 0 & 0 & 1/3 & 0\\ 0 & 0 & 0 & -2/3 \end{array}\right).$$

(D.2) $$\lambda_{1}=\left(\begin{array}{cccc} 0 & 0 & 0 & 0\\ 0 & 0 & 1 & 0\\ 0 & 1 & 0 & 0\\ 0 & 0 & 0 & 0 \end{array}\right)\;\lambda_{2}=\left(\begin{array}{cccc} 0 & 0 & 0 & 0\\ 0 & 0 & -i & 0\\ 0 & i & 0 & 0\\ 0 & 0 & 0 & 0 \end{array}\right)\;\lambda_{3}=\left(\begin{array}{cccc} 0 & 0 & 0 & 0\\ 0 & 1 & 0 & 0\\ 0 & 0 & -1 & 0\\ 0 & 0 & 0 & 0 \end{array}\right)$$

It seemed a priori difficult to fit these 4 dimensional matrices in the SU(2*/*1) framework and the quarks were listed in the original article of Ne’eman [1] as a counterexample to the SU(2*/*1) paradigm and left out in [2]. But soon after, in what could be described as the first success of the model, it was realized [7, 8] that such a representation had been found earlier by Scheunert, Nahm and Rittenberg [9]. The existence of the 4 dimensional quark representation is natural considering the isomorphism of the superalgebras SU(2*/*1) and OSp(2*/*2) which generalizes the well know Lie algebra isomorphisms of SU(2), Sp(2) and SO(3). The construction is simple. Since the electric charges of the up and down quarks (*u* and *d*) are respectively (2/3) and (−1/3), we can infer from the definition (B.9) of the photon matrix $\gamma =-\lambda_{6}^{2}$ the form of *λ*_6_ and the other odd matrices follow by commutation with SU(2). One obtains

(D.3) $$\lambda_{4}=\frac{1}{\sqrt{3}}\left(\begin{array}{cccc} 0 & 0 & -\sqrt{2} & 0\\ 0 & 0 & 0 & 1\\ \sqrt{2} & 0 & 0 & 0\\ 0 & 1 & 0 & 0 \end{array}\right)\lambda_{5}=\frac{1}{\sqrt{3}}\left(\begin{array}{cccc} 0 & 0 & i\sqrt{2} & 0\\ 0 & 0 & 0 & -i\\ i\sqrt{2} & 0 & 0 & 0\\ 0 & i & 0 & 0 \end{array}\right)\;\lambda_{6}=\frac{1}{\sqrt{3}}\left(\begin{array}{cccc} 0 & \sqrt{2} & 0 & 0\\ -\sqrt{2} & 0 & 0 & 0\\ 0 & 0 & 0 & 1\\ 0 & 0 & 1 & 0 \end{array}\right)\lambda_{7}=\frac{1}{\sqrt{3}}\left(\begin{array}{cccc} 0 & -i\sqrt{2} & 0 & 0\\ -i\sqrt{2} & 0 & 0 & 0\\ 0 & 0 & 0 & -i\\ 0 & 0 & i & 0 \end{array}\right)$$

A direct calculation shows that the quark matrices have the same commutators and anticommutators as the lepton matrices. In particular, we recognize the electric charge of the quarks in the diagonal photon matrix (B.9):

(D.4) $$\gamma =-\frac{1}{2}\left\{\lambda_{6},\lambda_{6}\right\}=-\frac{1}{2}\left\{\lambda_{7},\lambda_{7}\right\}=\frac{1}{2}\left(\lambda_{0}+\lambda_{3}\right)=\left(\begin{array}{cccc} 2/3 & 0 & 0 & 0\\ 0 & 2/3 & 0 & 0\\ 0 & 0 & -1/3 & 0\\ 0 & 0 & 0 & -1/3 \end{array}\right)$$

In Kac terminology, the quark representation is typical. Its Casimir operators (A.8) are diagonal with eigenvalues

(D.5) $$K_{2}=\frac{8}{9}I,\;K_{3}=-\frac{64}{27}I.$$

As in the case of the antileptons, the quark matrices are never given explicitly in the literature.

## E The OSp(2/2) neutral representation

It must be stressed that SU(2*/*1) does not predict that the hyper-charge of the left quark doublet is 1*/*3. This charge is a free parameter. One can construct an irreducible representation of SU(2*/*1) of arbitrary hyper-charge 1*/n* (*n* can be a complex number) using the same matrices *χ*, *λ*_1_, *λ*_2_, *λ*_3_ as before, selecting the desired values in *λ*_0_ and writing the corresponding *λ*_6_

(E.1) $$\lambda_{0}=\left(\begin{array}{cccc} (n+1)/n & 0 & 0 & 0\\ 0 & 1/n & 0 & 0\\ 0 & 0 & 1/n & 0\\ 0 & 0 & 0 & -(n-1)/n \end{array}\right),\;\lambda_{6}=\frac{1}{\sqrt{2n}}\left(\begin{array}{cccc} 0 & \sqrt{n+1} & 0 & 0\\ -\sqrt{n+1} & 0 & 0 & 0\\ 0 & 0 & 0 & \sqrt{n-1}\\ 0 & 0 & \sqrt{n-1} & 0 \end{array}\right).$$

The other odd generators are constructed by commutation with the even *λ*_*a*_ and have the same shape as for the quarks. When *n* = ±1, one of the 4 states (i.e. the right neutrino) decouples. When *n* = −1, we recover the lepton representation, when *n* = 1, the antileptons, when *n* = 3, the quarks. when *n* = −3, the antiquarks. The conclusion is that in SU(2*/*1) the electric charge is not quantized, but if the electric charge of the electron *e*^−^ is equal to the electric charge of the SU(2) lowering operator *λ*_1_ − *iλ*_2_, i.e. to the charge of the observed *W*^−^ Yang-Mills vector Boson, then the right neutrino decouples, it has no electric charge and no weak hyper-charge. Otherwise, in a quark like representation where the weak hyper-charge of the SU(2) doublet is neither 1 nor −1, the 2 right singlets must exist, and their electric charge must differ by 1 unit.

In the large *n* limit, we obtain the natural OSp(2*/*2) symmetric representation for which the doublet is SU(2) neutral.

(E.2) $$\lambda_{0}=\left(\begin{array}{cccc} 1 & 0 & 0 & 0\\ 0 & 0 & 0 & 0\\ 0 & 0 & 0 & 0\\ 0 & 0 & 0 & -1 \end{array}\right),\;\lambda_{6}=\frac{1}{\sqrt{2}}\left(\begin{array}{cccc} 0 & 1 & 0 & 0\\ -1 & 0 & 0 & 0\\ 0 & 0 & 0 & 1\\ 0 & 0 & 1 & 0 \end{array}\right).$$

In Kac terminology, the neutral representation is typical, its cubic super-Casimir operator (A.8) vanishes:

(E.3) $$K_{2}=I,\;K_{3}=0.$$

If we change variables and label the quadruplet representation by the hypercharge *y* of the SU(2) doublet (y = 1/n of equation (E.1)) we can label a family of representation by a vector {*y*_*i*_} giving the collection of its hypercharges. The standard model family (electron + 3 quarks) is labeled by the vector {−1,1*/*3,1*/*3,1*/*3}. By definition, the Adler triangle anomaly U(1)SU(2)SU(2) cancels out if Σ*y*_*i*_ = 0. In each representation *Y* = diagonal(*y* + 1,*y,y,y* − 1), hence *STr*(*Y* ^3^) = −6*y* and the U(1)^3^ triangle anomaly also cancels out if Σ*y*_*i*_ = 0. By inspection, the scalar anomalies (2.4) and (2.8) are also proportional Σ*y*_*i*_. We do not have a simple analytic proof but verified numerically that Δ(*ρ*) of equation (2.12) is linear in *y*. We conclude that any family such that Σ*y*_*i*_ = 0 is anomaly free. We have already presented three examples. The standard model family (electron + 3 quarks), the anti-family (positron + 3 antiquarks, the neutral family of Minahan, Ramond and Warner (a single OSp(2*/*2) neutral quark [21]). But a model with one quark with *y* = 2*/*3 and two quarks with *y* = −1*/*3 is also anomaly free. The electric charges of the *d*_*R*_ states would be (*y* − 1)*/*2, i.e. (−1/6, −2/3, −2/3).

## F The H/K Hermitian couplings

For completeness we give here explicitly the $\mu_{i}^{\pm }$ matrices which define the couplings of the *H* and *K* fields to the Fermions [15, 16]. In the lepton representation, the *μ*_*i*_ matrices are Hermitian, so $\mu_{i}^{-}=\lambda_{i}$ and $\mu_{i}^{+}=0$. In the positively charged antilepton representation, it is the opposite, $\mu_{i}^{-}=0$ and

(F.1) $$\mu_{4}^{+}=\left(\begin{array}{ccc} 0 & 0 & i\\ 0 & 0 & 0\\ -i & 0 & 0 \end{array}\right),\;\mu_{5}^{+}=\left(\begin{array}{ccc} 0 & 0 & 1\\ 0 & 0 & 0\\ 1 & 0 & 0 \end{array}\right),\;\mu_{6}^{+}=\left(\begin{array}{ccc} 0 & -i & 0\\ i & 0 & 0\\ 0 & 0 & 0 \end{array}\right),\;\mu_{7}^{+}=\left(\begin{array}{ccc} 0 & -1 & 0\\ -1 & 0 & 0\\ 0 & 0 & 0 \end{array}\right).$$

Finally, in the quark representation, the *μ*^−^ matrices coupled to the *H* field read

(F.2) $$\mu_{4}^{-}=\frac{1}{\sqrt{3}}\left(\begin{array}{cccc} 0 & 0 & 0 & 0\\ 0 & 0 & 0 & 1\\ 0 & 0 & 0 & 0\\ 0 & 1 & 0 & 0 \end{array}\right)\mu_{5}^{-}=\frac{1}{\sqrt{3}}\left(\begin{array}{cccc} 0 & 0 & 0 & 0\\ 0 & 0 & 0 & -i\\ 0 & 0 & 0 & 0\\ 0 & i & 0 & 0 \end{array}\right)\;\mu_{6}^{-}=\frac{1}{\sqrt{3}}\left(\begin{array}{cccc} 0 & 0 & 0 & 0\\ 0 & 0 & 0 & 0\\ 0 & 0 & 0 & 1\\ 0 & 0 & 1 & 0 \end{array}\right)\mu_{7}^{-}=\frac{1}{\sqrt{3}}\left(\begin{array}{cccc} 0 & 0 & 0 & 0\\ 0 & 0 & 0 & 0\\ 0 & 0 & 0 & -i\\ 0 & 0 & i & 0 \end{array}\right)$$

and the *μ*^+^ matrices coupled to the *K* field read

(F.3) $$\mu_{4}^{+}=\frac{1}{\sqrt{3}}\left(\begin{array}{cccc} 0 & 0 & i\sqrt{2} & 0\\ 0 & 0 & 0 & 0\\ -i\sqrt{2} & 0 & 0 & 0\\ 0 & 0 & 0 & 0 \end{array}\right)\mu_{5}^{+}=\frac{1}{\sqrt{3}}\left(\begin{array}{cccc} 0 & 0 & \sqrt{2} & 0\\ 0 & 0 & 0 & 0\\ \sqrt{2} & 0 & 0 & 0\\ 0 & 0 & 0 & 0 \end{array}\right)\;\mu_{6}^{+}=\frac{1}{\sqrt{3}}\left(\begin{array}{cccc} 0 & -i\sqrt{2} & 0 & 0\\ i\sqrt{2} & 0 & 0 & 0\\ 0 & 0 & 0 & 0\\ 0 & 0 & 0 & 0 \end{array}\right)\mu_{7}^{+}=\frac{1}{\sqrt{3}}\left(\begin{array}{cccc} 0 & -\sqrt{2} & 0 & 0\\ -\sqrt{2} & 0 & 0 & 0\\ 0 & 0 & 0 & 0\\ 0 & 0 & 0 & 0 \end{array}\right)$$

The $\mu_{i}^{\pm }$ matrices of the quarks are proportional to the non-zero $\mu_{i}^{\pm }$ of the leptons and antileptons, complemented by an extra line-column of zeroes, and the commutators with the even matrices $\left[\lambda_{a},X_{i}\right]=f_{ai}^{j}X_{j}$ have the same $f_{ai}^{j}$ structure constants when $X_{i}=\lambda_{i},\mu_{i}^{+},\mu_{i}^{-}$ in any representation.

## G The massive neutrino SU(2/1) indecomposable representation

The representations presented so far are irreducible. This means that all the states belonging to such a representation are equivalent in the sense that under the action of the superalgebra each of them generates all of them, or in other words each orbit covers the whole representation. In a Lie algebra, all finite dimensional representations are fully reducible. This means that they can be written as block diagonal matrices, where each block corresponds to an irreducible representation. But in a superalgebra, some finite dimensional representations are indecomposable. This means that the matrices are triangular, or in other words some orbits do not cover the whole representation. Rather than sketching the complete theory [10–12], we construct a few examples of SU(2*/*1) indecomposable representations relevant to the classifications of the elementary particles [13, 15, 16].

The simplest example is applicable to neutrinos. Consider (E.1) with *n* = −1, and let us call the four states *ν*_*R*_*/*(*ν*_*L*_,*e*_*L*_)*/e*_*R*_. The right neutrino, *ν*_*R*_ is SU(2) and U(1) neutral. This is experimentally correct, but we know that the neutrino has a very small but non-zero mass. Contrary to the case of a Lie algebra, it is possible in SU(2*/*1) to add a small scalar couplings of order *α* as follows.

(G.1) $$\chi =\left(\begin{array}{cccc} -1 & 0 & 0 & 0\\ 0 & 1 & 0 & 0\\ 0 & 0 & 1 & 0\\ 0 & 0 & 0 & -1 \end{array}\right),\;\lambda_{0}=\left(\begin{array}{cccc} 0 & 0 & 0 & 0\\ 0 & -1 & 0 & 0\\ 0 & 0 & -1 & 0\\ 0 & 0 & 0 & -2 \end{array}\right)\;\lambda_{1}=\left(\begin{array}{cccc} 0 & 0 & 0 & 0\\ 0 & 0 & 1 & 0\\ 0 & 1 & 0 & 0\\ 0 & 0 & 0 & 0 \end{array}\right)\;\lambda_{2}=\left(\begin{array}{cccc} 0 & 0 & 0 & 0\\ 0 & 0 & -i & 0\\ 0 & i & 0 & 0\\ 0 & 0 & 0 & 0 \end{array}\right)\;\lambda_{3}=\left(\begin{array}{cccc} 0 & 0 & 0 & 0\\ 0 & 1 & 0 & 0\\ 0 & 0 & -1 & 0\\ 0 & 0 & 0 & 0 \end{array}\right)\;\lambda_{4}=\left(\begin{array}{cccc} 0 & 0 & 0 & 0\\ 0 & 0 & 0 & 1\\ \alpha & 0 & 0 & 0\\ 0 & 1 & 0 & 0 \end{array}\right)\;\lambda_{5}=\left(\begin{array}{cccc} 0 & 0 & 0 & 0\\ 0 & 0 & 0 & -i\\ i\alpha & 0 & 0 & 0\\ 0 & i & 0 & 0 \end{array}\right)\;\lambda_{6}=\left(\begin{array}{cccc} 0 & 0 & 0 & 0\\ -\alpha & 0 & 0 & 0\\ 0 & 0 & 0 & 1\\ 0 & 0 & 1 & 0 \end{array}\right)\;\lambda_{7}=\left(\begin{array}{cccc} 0 & 0 & 0 & 0\\ -i\alpha & 0 & 0 & 0\\ 0 & 0 & 0 & -i\\ 0 & 0 & i & 0 \end{array}\right)$$

A direct calculation shows that these modified matrices have the same commutators and anticommutators as the lepton matrices. The even matrices are equivalent to (B.1), (B.3) with an additional first line and first column of zeroes, meaning that *ν*_*R*_ remains SU(2)U(1) neutral. The last line and last column of the odd matrices reproduce (B.5). The new *α* terms occur in the first column, but are omitted from the first line. Thus, each matrix is block triangular. There are two highest vectors annihilated by all the raising operators *ν*_*R*_ and *ν*_*L*_. Since (*λ*_6_ − *iλ*_7_)*ν*_*R*_ = −2*αν*_*L*_, the orbit of *ν*_*R*_ is the whole representations whereas the orbits of the three other states does not cover *ν*_*R*_. Notice that there is a single free parameter, because the four terms in *α* are linked bu the action of SU(2). To verify that we still have a representation of the superalgebra, we just need to compute one anticommutator, say {*λ*_4_,* λ*_6_} and check that the lower left corner element vanishes. As shown in [16], if *H*^6^ acquires a vacuum expectation value *h*, the neutrino acquires a mass of order *αh*. A contrario, if we try to apply this mechanism to a Lie algebra and consider the same matrices, we would need to compute the commutator [*λ*_4_,* λ*_6_], and we would generate a non-zero value in the lower-left corner, verifying on this simple example that we cannot construct a four-dimensional indecomposable representation of SU(3).

## H The three generations SU(2/1) indecomposable representation

In our second example, we show that in SU(2*/*1), it is possible to mix several copies of the same irreducible representation, explaining the existence of the three generations of leptons and quarks labeled by the electron, the muon and the tau. Relative to a Lie algebra, the peculiarity is that we can construct a representation were the maximal commuting Cartan subalgebra cannot be diagonalized. Consider the 8×8 block triangular matrices

(H.1) $${\text{Λ}}_{a}=\left(\begin{array}{cc} \lambda_{a} & 0\\ \theta {\bar{\lambda }}_{a} & \lambda_{a} \end{array}\right),\;{\text{Λ}}_{i}=\left(\begin{array}{cc} \lambda_{i} & 0\\ \theta {\bar{\lambda }}_{i} & \lambda_{i} \end{array}\right)$$

where *θ* is a arbitrary mixing angle which can be thought of as a parametrization of the Cabbibo angle [15]. The *λ* are the quark matrices given in equations (D.3), (D.4), ${\bar{\lambda }}_{0}=4\sqrt{2}/3Id$, where *Id* is the 4 × 4 identity matrix, ${\bar{\lambda }}_{a}=0,\;a=1,2,3$, and the off diagonal odd matrices read

(H.2) $${\bar{\lambda }}_{4}=\frac{1}{\sqrt{3}}\left(\begin{array}{cccc} 0 & 0 & -1 & 0\\ 0 & 0 & 0 & \sqrt{2}\\ 1 & 0 & 0 & 0\\ 0 & \sqrt{2} & 0 & 0 \end{array}\right),{\bar{\lambda }}_{5}=\frac{1}{\sqrt{3}}\left(\begin{array}{cccc} 0 & 0 & i & 0\\ 0 & 0 & 0 & -i\sqrt{2}\\ i & 0 & 0 & 0\\ 0 & i\sqrt{2} & 0 & 0 \end{array}\right)\;{\bar{\lambda }}_{6}=\frac{1}{\sqrt{3}}\left(\begin{array}{cccc} 0 & 1 & 0 & 0\\ -1 & 0 & 0 & 0\\ 0 & 0 & 0 & \sqrt{2}\\ 0 & 0 & \sqrt{2} & 0 \end{array}\right),{\bar{\lambda }}_{7}=\frac{1}{\sqrt{3}}\left(\begin{array}{cccc} 0 & -i & 0 & 0\\ -i & 0 & 0 & 0\\ 0 & 0 & 0 & -i\sqrt{2}\\ 0 & 0 & i\sqrt{2} & 0 \end{array}\right)$$

By inspection, one can verify that these eight matrices have the same commutators as the quark matrices and thus form an eight dimensional indecomposable representation of SU(2*/*1). This representation was first proposed, up to notations, in [13], and is implicit in [10, 12], but is not included in [11] who only analyse the case where the Cartan subalgebra is diagonal. We are grateful to Coquereaux, Quella, Schomerus and Sorba for clarifying this point. The construction can be extended to three generation using 12×12 block triangular matrices.

(H.3) $$\text{Λ}=\left(\begin{array}{ccc} \lambda & 0 & 0\\ \bar{\lambda } & \lambda & 0\\ \nu & \bar{\lambda } & \lambda \end{array}\right),\;\nu_{6}=\frac{1}{2\sqrt{3}}\left(\begin{array}{cccc} 0 & \sqrt{2} & 0 & 0\\ -\sqrt{2} & 0 & 0 & 0\\ 0 & 0 & 0 & -5\\ 0 & 0 & -5 & 0 \end{array}\right)$$

Along the diagonal, we have three identical copies of the quark representation. Just below the diagonal, we have 2 copies of the previous ‘Cabbibo’ construction. The *ν*_*a*_ matrices again vanish for *a* = 1,2,3, and *ν*_0_ = 2*Id* is again proportional to the identity. The matrix *ν*_6_ is constrained. The matrices *ν*_*i*_,*i* = 4,5,7 are deduced from *ν*_6_ by commutation with the SU(2) generators. One can then introduce a parametrization *α,β,γ* of the Cabbibo-Kobayashi-Maskawa mixing angles [15],

(H.4) $$\text{Λ}=\left(\begin{array}{ccc} \lambda & 0 & 0\\ \alpha \bar{\lambda } & \lambda & 0\\ \gamma \nu & \beta \bar{\lambda } & \lambda \end{array}\right)$$

and solve two linear equations to adjust the scale of *ν*_0_ and the entries in *ν*_6_.

The construction cannot be extended to four generations, because there would be to many constraints in the lower left corner. In physics terminology, SU(2*/*1) can describe the scalar mixing of 3 generations of quarks or leptons using a single indecomposable twelve dimensional representation but does not allow a fourth generation.

## I The Adler-Bell-Jackiw vector anomaly

Having recognized that the smallest representation of the SU(2*/*1) superalgebra correctly describes the quantum numbers of the electrons and the quarks, we have solved the classic static classification problem. We now consider the quantum dynamic problem and wonder if the adjoint representation of SU(2*/*1) can describe the vectors Bosons of the standard model. As usual, we associate a real Yang-Mills vector field $A_{\mu }^{a}$ to each even generator *λ*_*a*_, and postulate that its couplings to the Fermion fields are given by the chiral Weyl-Dirac Lagrangian

(I.1) $$L_{\psi }^{\left(A\right)}={\bar{\left(\psi_{R}\right)}}_{L}\sigma^{\mu }\left(\partial_{\mu }+A_{\mu }^{a}\lambda_{a}\right)\psi_{R}+{\bar{\left(\psi_{L}\right)}}_{R}{\bar{\sigma }}^{\mu }\left(\partial_{\mu }+A_{\mu }^{a}\lambda_{a}\right)\psi_{L}$$

where the spin-one Pauli matrices *σ* map the right spinors on the left spinors and the $\bar{\sigma }$ matrices map the left spinors on the right spinors. In Minkowski space they can be represented as:

(I.2) $$\sigma_{0}=\left(\begin{array}{cc} 1 & 0\\ 0 & 1 \end{array}\right),\;\sigma_{1}=\left(\begin{array}{cc} 0 & 1\\ 1 & 0 \end{array}\right),\;\sigma_{2}=\left(\begin{array}{cc} 0 & -i\\ i & 0 \end{array}\right),\;\sigma_{3}=\left(\begin{array}{cc} 1 & 0\\ 0 & -1 \end{array}\right)\;{\bar{\sigma }}_{0}=\left(\begin{array}{cc} -1 & 0\\ 0 & -1 \end{array}\right),\;{\bar{\sigma }}_{1}=\left(\begin{array}{cc} 0 & 1\\ 1 & 0 \end{array}\right),\;{\bar{\sigma }}_{2}=\left(\begin{array}{cc} 0 & -i\\ i & 0 \end{array}\right),\;{\bar{\sigma }}_{3}=\left(\begin{array}{cc} 1 & 0\\ 0 & -1 \end{array}\right)$$

They are Hermitian and satisfy the chiral Clifford-Weyl relations

(I.3) $$\sigma_{\mu }{\bar{\sigma }}_{\nu }+\sigma_{\nu }{\bar{\sigma }}_{\mu }=2g_{\mu \nu }I_{L}\;{\bar{\sigma }}_{\mu }\sigma_{\nu }+{\bar{\sigma }}_{\nu }\sigma_{\mu }=2g_{\mu \nu }I_{R}$$

where $g_{\mu \nu }=g^{\mu \nu }$ denotes the diagonal Minkowski metric (−1,1,1,1). Importantly, if we compute the trace of the product of four *σ* matrices we find a tensor with mixed symmetry

(I.4) $$Tr\left(\sigma_{\mu }{\bar{\sigma }}_{\nu }\sigma_{\rho }{\bar{\sigma }}_{\sigma }\right)=2\left(g_{\mu \nu }g_{\rho \sigma }-g_{\mu \rho }g_{\nu \sigma }+g_{\mu \sigma }g_{\nu \rho }+i\epsilon_{\mu \nu \rho \sigma }\right)\;Tr\left({\bar{\sigma }}_{\mu }\sigma_{\nu }{\bar{\sigma }}_{\rho }\sigma_{\sigma }\right)=2\left(g_{\mu \nu }g_{\rho \sigma }-g_{\mu \rho }g_{\nu \sigma }+g_{\mu \sigma }g_{\nu \rho }-i\epsilon_{\mu \nu \rho \sigma }\right)$$

where the *g* terms are symmetric, and *ϵ* is fully antisymmetric in *μνρσ* with *ϵ*_0123_ = 1.

We know that the Yang-Mills theory is multiplicatively renormalizable but in the presence of chiral Fermion there is an obstruction visible in the evaluation of the self interaction of the vectors. The classical vertex is given by the term cubic in *A* inside $L=-1/4F_{\mu \nu }^{2}$. It is proportional to $f_{abc}\partial_{\mu }A_{\nu }^{a}A_{\mu }^{b}A_{\nu }^{c}$. We must verify that the divergent part of the one-loop quantum correction has the same tensorial structure as the classical term, so that, following Feynman’s prescription, the divergence can be absorbed in a redefinition of the coupling constant. In a non-chiral Yang-Mills theory, this is always true. But as found by Adler [5] and Bell-Jackiw [6], there is a subtle problem in the presence of chiral Fermions. The calculation is at the same time complicated and well known, so we only present a few crucial points. Consider the four distinct diagrams with 3 external vectors $A_{\mu }^{a}A_{\nu }^{b}A_{\rho }^{c}$ hitting a chiral Fermion loop.

First, each loop involves an integration over the momentum of the Fermions. The sign of the Fermion propagators depends on the orientation of the loop. Since each diagram contains 3 propagators, the signs are flipped when we reverse the orientation of the loops. The sum is antisymmetric under the simultaneous exchange of (*b,ν*) with (*c,ρ*).

Next, one must trace over the six Pauli matrices, 3 coming from the vertices, 3 from the propagators. For the left Fermion loops, the propagators use the $\bar{\sigma }$ and the vertex use the *σ*, and vice-versa for the right Fermion loops. Thus in (I.4) the sign of the $\epsilon_{\mu \nu \rho \sigma }$ term depends on the chirality of each loop.

Finally, we trace over the *λ* matrices. We get 2 terms. The first term from (I.4) receives the same sign from the left and right Fermions and is symmetric in *νρ* hence skew in (*bc*) yielding $Tr\left(\lambda_{a}{\left[\lambda_{b},\lambda_{c}\right]}_{-}\right)$. In any representation of a Lie algebra, this trace (A.6) is proportional to the structure constants of the Lie algebra, as hoped for. However, the second term from (I.4) with the *ϵ* Lorentz structure is skew in *νρ* hence symmetric in (*bc*) but as the overall sign depends on the chirality of the Fermion loop, this term involves a supertrace. It has the wrong tensorial structure, should vanish, and its matrix dependent part reads

(I.5) $$STr\left(\lambda_{a}{\left\{\lambda_{b},\lambda_{c}\right\}}_{+}\right)=0,$$

This term (1.2) is known as the triangle anomaly. It matches the even part (A.7) of the cubic super-Casimir tensor of the SU(2*/*1) superalgebra.

As discovered by Bouchiat, Iliopoulos and Meyer [20] in 1972 for the group SU(2)U(1), this term is non zero on the leptons and the quarks but the sum of the 2 contributions vanishes when we have 3 quarks for every lepton. This implied the existence of a pair of quark flavors for the electron (the up and down quarks), and a second pair (strange and charm) associated to the muon at a time when the charm quark was not yet directly observed. It also implied the existence of the top quark after the discovery of the *τ* lepton and bottom quark. For us, the occurrence of the chiral supertrace indicates the need for a chiral superalgebraic description of the electroweak interactions.

There is a second solution, discussed in the elegant note of Minahan, Ramond and Warner in 1990 [21], which also has a neat SU(2*/*1) interpretation. In the large *n* limit, the SU(2*/*1) neutral quarks of the previous section (E.2), with electric charge ±1*/*2, have a vanishing *K*_3_ Casimir operator (E.3) and are anomaly free by themselves, hence they require no leptons.

Finally, from the point of view of SU(2*/*1), if we knew nothing of the strong interactions, we could also accept as a solution of the Adler-Bell-Jackiw constraints, five SU(5) quarks with electric charge 3*/*5 and −2*/*5 or more generally *n* SU(*n*) quarks of electric-charge (*n* + 1)*/*2*n* and −(*n* − 1)*/*2*n*. In the SU(5) grand unified theory, which breaks down to SU(3)SU(2)U(1), the charge of the quarks are automatically 2*/*3 and −1*/*3, but this is a tautology, because if the strong interactions were described by an SU(5) group, we could as well have chosen an SU(8) grand unified theory breaking down to SU(5)SU(2)U(1) and predict the charge of the quarks to be 3*/*5 and −2*/*5.
